# A *Nano Letters* Journey

**DOI:** 10.1021/acs.nanolett.5c03678

**Published:** 2025-09-10

**Authors:** Dmitry Baranov

**Affiliations:** † Division of Chemical Physics and NanoLund, Department of Chemistry, 5193 Lund University, P.O. Box 124, Lund SE-221 00, Sweden

The journals we read and learn from become as influential as the mentors who guide and inspire us. It is especially true when professional and personal lives intertwine, as they often do for scientists. *Nano Letters* has been one such journal for me. That is why it feels especially profound to join the Nano Letters Early Career Board and have the opportunity to contribute to this foundational journal. What started 22 years ago as a chance encounter with a *Nano Letters* paper has now come full circle.

In the late fall of 2003, as a freshman student in Moscow, I stopped by a newspaper kiosk on my way to physical chemistry class to pick up a copy of *Lomonosov*, a popular science magazine at the time, known for its translations from *New Scientist*. In the issue I grabbed that morning was an article discussing how a carbon nanotube filled with water molecules could act as an electrical cable, conducting electricity using protons instead of electrons. I did not yet know what a nanotube was, or what a proton wire meant, but the unknown packaged in an engaging narrative captivated me. Years later, I traced the origin of that speculative piece to a 2003 *Nano Letters* paper by R. Jay Mashl and colleagues.^1^ Their molecular dynamics simulations showed that water molecules could align into an ice-like order inside nanotubes of a specific diameter, hinting at a pathway for proton conductivity.

Coinciding with that article, I had to choose a research group for a semester-long course in inorganic chemistry. When asked by the course coordinator where I would like to do the project, I asked if there was a lab working on nanomaterials. That led me to the Chemistry of Nanomaterials Laboratory, headed by Sergey Gubin at the Kurnakov Institute of General and Inorganic Chemistry, where I spent several years as an undergraduate researcher. The work involved synthesizing composites of organic polymers and nanoparticles via the thermal decomposition of metal salts in polymer melts. The institute had broadband Internet, but lacked subscriptions to journals from ACS Publications. A senior colleague, Sergey Shchepinov, made regular trips to the Academic Library and returned with printouts and PDFs of recent publications in nanoscience. Thanks to his efforts, we were able to read the latest papers from *Nano Letters*, among other journals.

In the mid-2000s, III–V compound semiconductor nanowires were breaking new ground, and one of the top groups was led by Lars Samuelson at what is now NanoLund. Their electron microscopy images of atomic interfaces between different semiconductors, in my eyes, were a quintessential showcase of the possibilities brought by nanotechnology.^2^ To a synthetic chemist with modest lab facilities, epitaxy and vapor–liquid–solid growth methods and high-resolution electron microscopy were out of reach. So, learning about an alternative approach was a turning point for me.

In 2008, I read a study in *Nano Letters* from the National Nanotechnology Laboratory (NNL) in Lecce, Italy, describing the colloidal synthesis of nanoscale cobalt-titania heterostructures.^3^ The electron microscopy images of atomic interfaces between such dissimilar materials, synthesized via a hot-injection-inspired method, were incredible and very much like those from epitaxially grown nanostructures. Yet the synthesis was accessible and understandable, as by then, I had some hands-on experience with the thermal decomposition of cobalt carbonyl and titanium alkoxides. That relation made the exotic nanoworld feel a bit more reachable. I was fortunate to get an opportunity for a research stay in Lecce, and a work from that time with Liberato Manna at NNL and IIT Genova led to my first first-author paper in *Nano Letters*.^4^

*Nano Letters* continued to influence my journey in graduate school. In 2011, I began my Ph.D. at CU Boulder in David Jonas’s group, which specializes in ultrafast laser spectroscopy. The intersection of nanochemistry and spectroscopy contributed to explaining the line shape of a 2D electronic spectrum of colloidal PbSe quantum dots.^5^ That experience of bringing together different research areas for deeper insights into a nanomaterial continues to guide my current work. It is fair to say that one of the most recognizable characteristics of an article from *Nano Letters* is interdisciplinarity, delivered in a concise and impactful form.

Nearing graduation and facing another crossroads, I came across a report from Maksym Kovalenko’s group about colloidal perovskite nanocrystals.^6^ Reading it early on and seeing bright, luminescent colloids in a neighboring lab soon after solidified my motivation to seek postdoctoral work with perovskites. That brought me back to Italy in 2017 and set a trajectory toward independent research. After several productive years at IIT Genova, I started the Nanochemistry and Spectroscopy group at Lund University at the end of 2022, focusing on collective properties of nanocrystals and related materials.

The access to scientific knowledge has changed beyond recognition over the past two decades. Ubiquitous Internet, open-access publishing, and rapid dissemination via social media have made research more available than ever, but also brought information overload. What endures is the power of science to inspire and a chance that a single paper can set someone on a path in research (Figure 1). That is why it is so thrilling to join the *Nano Letters* Early Career Board and hopefully add my two cents to the journey of shaping the future of nanoscience.

**Figure 1:**
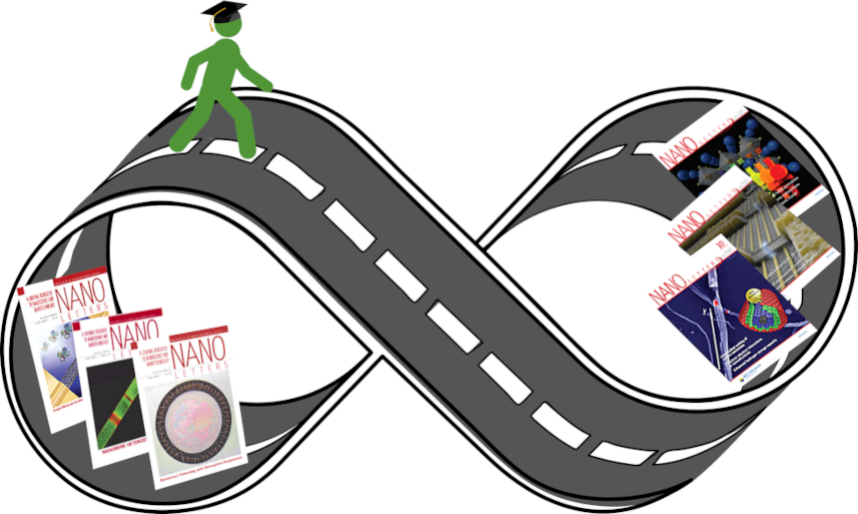
Möbius road illustrates the researcher’s path, marked by issues of *Nano Letters* that feature papers cited in this Viewpoint. Cover images reproduced from *Nano Letters*. Copyright American Chemical Society.
